# Genome-Wide Association Study for Individual Primal Cut Quality Traits in Canadian Commercial Crossbred Pigs

**DOI:** 10.3390/ani15121754

**Published:** 2025-06-13

**Authors:** Zohre Mozduri, Graham Plastow, Jack Dekkers, Kerry Houlahan, Robert Kemp, Manuel Juárez

**Affiliations:** 1 Livestock Gentec Centre, Department of Agricultural, Food and Nutritional Science, University of Alberta, Edmonton, AB T6G 2E1, Canada; mozduri@ualberta.ca (Z.M.); plastow@ualberta.ca (G.P.); 2 Department of Animal Science, Iowa State University, Ames, IA 50011, USA; jdekkers@iastate.edu; 3 Genesus Genetic Technology Inc., Winnipeg, MB R3P 0H4, Canada; khoulahan@genesus.com; 4 RAK Genetic Consulting Ltd., Lethbridge, AB T1K 6A9, Canada; bob.kemp@gmail.com; 5 Lacombe Research and Development Centre, Agriculture and Agri-Food Canada, Lacombe, AB T4L 1W1, Canada

**Keywords:** SNPs, pork quality, primal fat, fat metabolism, whole genome sequencing

## Abstract

This study identified genomic variants and potential candidate genes associated with 11 primal cut traits (back fat, belly fat, total fat, loin fat, ham fat, picnic fat, butt fat, loin intramuscular fat content, ham side fat, shoulder dorsal fat, and belly side fat thicknesses) in Canadian commercial crossbred pigs. Genome-wide association studies using whole genome sequencing data were conducted using genotyping data from 1118 commercial crossbred pigs. This analysis revealed multiple QTLs across chromosomes SSC1, 2, 3, 6, 7, 9, 14, 15, and 17, associated with fat traits. Notably, an SNP at position 160,230,075 bp on SSC1 was significantly associated with multiple fat traits, including belly fat, butt fat, ham fat, loin fat, picnic fat, and side fat. Common genes in windows associated with multiple traits, such as *MC4R*, *RNF152*, and *CDH20* were shared across these traits, suggesting pleiotropic effects. Some of the QTLs were near previously identified QTLs or candidate genes that have been reported to be linked to meat quality traits associated with backfat and intramuscular fat. Other candidate genes identified in the study include *TNFRSF11A*, *LEPR*, and genes from the *SERPINB* family, highlighting their roles in fat deposition and composition. Additional candidate genes were also implicated in regulation of fat metabolism, adipogenesis, and adiposity. These findings offer valuable insights into the genetic architecture of fat traits in pigs, which could inform breeding strategies aimed at improving the pork quality.

## Simple Summary

Primal cut traits (back fat, belly fat, total fat, loin fat, ham fat, picnic fat, butt fat, loin intramuscular fat content, ham side fat, shoulder dorsal fat, and belly side fat thicknesses) are important characteristics influencing carcass composition and pork quality. Understanding the genetic basis of these traits provides valuable insights for genetic improvement in pigs. In this study, genome-wide association analyses were conducted using whole-genome sequencing data from 1118 Canadian commercial crossbred pigs to identify genomic regions associated with eleven primal fat traits. Several significant QTLs were detected across chromosomes SSC1, SSC2, SSC3, SSC6, SSC7, SSC9, SSC14, SSC15, and SSC17. A notable SNP on SSC1 was found to be associated with multiple fat traits, suggesting pleiotropic effects. Candidate genes such as *MC4R*, *RNF152*, *CDH20*, *TNFRSF11A*, and *LEPR* were identified, many of which are involved in fat metabolism and adipogenesis. These findings contribute to a deeper understanding of the genetic architecture of fat traits and offer potential for improving pork quality and carcass composition through genomic selection strategies.

## 1. Introduction

Swine breeders have traditionally prioritized enhancements in growth performance and lean meat yield through the application of genetic selection techniques [1]. Carcass and meat quality are critical to the pork industry, affecting both domestic and export markets, as well as overall profitability. These quality attributes have a direct impact on consumer purchasing behavior, resulting in a recent amplified focus on quality considerations within the realm of pork production [2]. Both genetic and environmental factors are key in shaping pork quality, with carcass and meat quality traits showing moderate to high heritability [3]. A comprehensive understanding of the genetic underpinnings of these traits is imperative for their enhancement using selective breeding. Genomic regions identified and validated for their influence on meat quality can be incorporated into genomic selection, enhancing selection accuracy, and speeding up genetic gains.

Backfat depth is a key indicator of overall fat content in live pigs [4]. Intramuscular (IMF) and subcutaneous fat are crucial for flavor, palatability, and quality in both fresh and processed pork [5]. Modern breeding has led to leaner pigs with more efficient muscle growth but lower fat quality, affecting meat processing and sensory properties [6]. Pork belly, a valuable cut with high fat content (30–60%), is an economically important part of the carcass [7]. As demand for high-quality pork bellies grows, understanding and predicting belly composition is becoming increasingly important [8]. While ham is less valuable per kg, it constitutes 23.5% of the carcass weight, making it significant to overall carcass value [9]. Finally, breeding for optimized fat content and composition is important for enhancing pork quality [10,11].

Canadian pork producers are in the process of modifying their breeding programs to prioritize pork quality and carcass traits in response to global market demands [12]. Recent advances in sequencing technologies have allowed the identification of many genes, quantitative trait loci (QTLs), and single nucleotide polymorphisms (SNPs) associated with pig carcass traits [1,13]. Genome-wide association studies (GWAS) utilizing sequence data have proven effective in pinpointing genomic regions associated with traits of interest, including QTLs [14]. The decrease in genome resequencing costs has facilitated the broad use of whole-genome sequencing (WGS) in GWAS, enabling more precise identification of QTL regions [15]. WGS provides an opportunity to enhance the detection of genetic regions affecting pork quality by capturing all significant SNPs, including rare variants [13]. Several studies have identified potential candidate genes associated with traits such as loin muscle area, backfat, and IMF.

Genome-wide association studies have identified several candidate genes associated with fat deposition and lean meat traits in pigs. For example, using whole-genome sequencing data, *SHANK*2 has been identified as a strong candidate gene associated with backfat thickness [1]. Similarly, Gozalo-Marcilla et al. (2021) reported several major genes involved in fat deposition, such as *MC4R*, *IGF2*, and *LEPR*, through large-scale genome-wide association studies for backfat thickness in pigs [16]. In their study, *MC4R* on SSC1 and *IGF2* on SSC2 were identified as key candidate genes contributing to the genetic variance of this trait [16]. This study aimed to identify new regions and to validate previously reported QTLs and candidate genes associated with primal cut traits, providing comprehensive insights into the genetic architecture of fat deposition in Canadian commercial crossbred pigs.

## 2. Material and Methods

### 2.1. Statement of Ethics

The experimental procedures were approved by the Animal Care Committee of the Agriculture and the Agri-Food Canada Lacombe Research and Development Center (AAFC-LRDC) under protocol #202204, following the principles and guidelines of the Canadian Council on Animal Care.

### 2.2. Animal Population and Phenotypes

Phenotypic data for carcass and meat quality traits were collected on 1118 commercial crossbred pigs (498 females and 620 males), which were offspring of Duroc sires and F1 hybrid (Landrace × Large White) dams, sourced from Genesus Genetic Technology (London, ON, Canada). Animals were raised under standard commercial practices to ~125 kg live weight and then shipped to the AAFC-LRDC federally inspected abattoir for slaughter. During bleeding, blood samples were collected for genomic analysis. Carcass weight and backfat depth between the 3rd and 4th last ribs were recorded using a grading probe. Carcasses were divided into primal cuts based on the International Meat Purchase Specifications for pork (IMPS, 2014) and fat content was measured using dual energy x-ray absorptiometry (DEXA) [17].

Total carcass fat was calculated from all primal cuts. A loin chop at the grading site and backfat samples were collected for further analysis. Intramuscular fat (IMF) content was measured using the Smart Trac Fat Analyzer Model 907,955 (CEM Corporation, Matthews, NC, USA). A 5 g backfat sample from the shoulder of each carcass was collected and stored at −80 °C for subsequent fatty acid analysis, following the protocol described previously [18]. Images of the primal cut surfaces (ham, shoulder, and belly) were captured for image analysis. Measurements included dorsal and side ham fat thickness, dorsal shoulder fat thickness, fat area percentage of ham and shoulder surfaces, and belly side fat, all assessed according to previously published protocols [19,20,21].

### 2.3. Genomic Analyses

Blood samples were genotyped for 36,566,734 SNP markers using SkimSEEK™ (a low-pass sequencing method imputed up to whole-genome sequence; Neogen^®^, Edmonton, AB, Canada). Quality control measures were applied to exclude SNPs from the whole-genome sequencing data using PLINK 2.00a3.6 [22] based on the following criteria: minor allele frequency (MAF) < 0.01, genotyping rate < 0.01, sample genotyping rate < 0.1 (mind), and deviation from Hardy–Weinberg equilibrium with a *p*-value < 1 × 10^−6^. Only SNPs located on autosomal chromosomes were included in this study. No animals or variants were excluded due to missing genotype data, so imputation was not necessary. After quality control, data on 1118 pigs and 18,911,793 SNPs were retained for further analysis.

### 2.4. Statistical Analysis

Both fixed and random effects were assessed for inclusion in the statistical model for analysis of phenotypes by fitting a linear mixed model to the data using the lmer4 package (version 1.1.35) in R software, employing REML or maximum likelihood estimation. Commercial carcass weight was included as a covariate in the model for all traits. The model employed for the GWAS was a single-marker mixed linear association model (MLMA), implemented in GCTA version 1.26.0 [23].

The model is represented by:

***y* = 1***µ***+ *Xb* + *Zu* + *W*_1_*c*_1_ + *W*_2_*c*_2_ + *e***

where *y* denotes the vector of phenotypes for all (*n*) animals; *µ* is the overall mean; *b* is a vector of (***p***) fixed effects (including the additive effect of SNP genotype, sex, boar line and boar group); ***X*** is the incidence matrix of fixed effects (*n* × *p*) linking the records in y to the fixed effects in b in which SNP genotypes are coded as 0, 1, or 2; *u* is a vector of polygenic random effects; ***Z*** is an incidence matrix that relates records to the polygenic effects; c_1_ and c_2_ are vectors of q levels (*q* × 1) of random effects of contemporary group (slaughter year and kill number) and common litter, and e represents a vector of random residual terms (*n* × 1); *W*_1_ and *W*_2_ represent design matrices (*n* × *q*_1_ and *n* × *q*_2_), relating to the records in y with the random effects in **c_1_** and **c_2_**, respectively. It is assumed that ***u*** ~ *N*(0, **GRM** *σ*^2^*_u_*) and ***e*** ~ *N*(0, *I σ*^2^*_e_*), where GRM is the genomic relationship matrix; and σ^2^_u_ and σ^2^_e_ represent the additive genetic and residual variances, respectively.

To correct for multiple testing, we employed the simple method proposed by Gao et al. (2008) [24]. This method considers the degree of linkage disequilibrium (LD) among SNPs in order to calculate the effective number of independent tests. For each chromosome, a correlation matrix is created using the composite LD correlation, and 18 values are then derived from a principal component analysis of the composite matrix. The number of principal components needed to explain 99% of the variance of genotypes on that chromosome is its effective numbers of SNPs. The total effective number of SNPs (Meff) is calculated by summing the effective numbers for each chromosome. *p*-values from SNP association tests were then adjusted for multiple comparisons using the Šidák correction with Meff. The adjusted *p*-value is computed as adjusted *p*-value = 1 − (1 − *p*-value)^Meff^ [25]. The qqman R package (version 0.1.9) was used to create Manhattan and Q–Q plots [26] for visualizing GWAS results, with the Manhattan plot highlighting significant associations by setting a genome-wide significance threshold at *p* < 2.62 × 10^−7^ and using color-coded chromosomes.

### 2.5. Post-GWAS Analyses

We utilized Ensembl BioMart (https://bioconductor.org/packages/biomaRt/, accessed on 15 June 2024) with the *Sus scrofa* 11.1 genome assembly (https://www.ncbi.nlm.nih.gov/datasets/genome/GCF_000003025.6/, accessed on 15 June 2024) to identify candidate genes associated with the SNPs in the significant regions, as well as neighboring SNPs located within 0.5 Mbp upstream and downstream of these regions. The choice of a 0.5 Mbp distance was based on the observation that the average linkage disequilibrium (LD) in commercial pig breeding populations drops below 0.3 when SNPs are more than 0.5 Mb apart [27]. Consequently, the significant window was defined as 0.5 Mb upstream and downstream of the significant SNPs identified in the GWAS that were located within this range. Additionally, the proportion of variance explained by each significant SNP was the amount of genetic variance reduced after adding the significant SNP to the model in GCTA version 1.26.0 [23], divided by the phenotypic variance. Finally, we used the UpSetR package in R, a customizable tool for data exploration and set visualization, to display the number of common SNPs and overlapping genes shared among the 11 traits in our GWAS analysis, providing a clear alternative to complex Venn diagrams when working with multiple datasets [28].

To explore the relationships between genes and primal cut traits based on shared SNPs, a binary gene–trait matrix was constructed and visualized as a heatmap using R (version 4.4.3). Data preparation and transformation were performed using the tidyverse package. A binary matrix was created in which rows represented genes and columns represented traits, with “1” indicating the presence of a shared SNP-based association and “0” indicating its absence. To highlight pleiotropic effects, only genes associated with two or more traits were included. The heatmap was generated using the pheatmap package. This approach enabled a concise visual summary of key genes involved in multiple traits. To evaluate overlapping genetic architecture across multiple fat traits, we conducted a meta-analysis of GWAS summary statistics for loin fat and butt fat, which were analyzed using a univariate linear mixed model. These traits were chosen based on overlapping QTL regions observed in single-trait GWAS and their relevance to subcutaneous carcass fat distribution. We used the METAL software (version 2011-03-25) developed by the Center for Statistical Genetics (University of Michigan; available at https://csg.sph.umich.edu/abecasis/Metal/download/, accessed on 11 June 2025). The analysis was conducted using the inverse-variance weighting scheme, incorporating effect sizes (b), standard errors (SE), *p*-values, and allele information for each SNP. The final results were filtered using a genome-wide significance threshold of *p* < 2.62 × 10⁻^7^. To further support the selection of candidate SNPs associated with multiple traits, we performed a linkage disequilibrium (LD) analysis using PLINK (version 1.9). SNPs identified as being linked to three or more primal cut traits in the GWAS were extracted, and pairwise LD was computed. SNP pairs with strong correlation (r^2^ > 0.8) were retained for interpretation. Additionally, functional enrichment analysis was conducted using the DAVID Functional Annotation Tools (https://davidbioinformatics.nih.gov/tools.jsp, accessed on 11 June 2025), employing the Benjamini–Hochberg procedure to adjust for multiple comparisons and control the false discovery rate (FDR) [29]. The significance threshold for enrichment was set at false discovery rate (FDR) < 0.1, adjusted using the Benjamini–Hochberg procedure to account for multiple testing.

## 3. Results and Discussion

### 3.1. Descriptive Statistics

Descriptive statistics for the 11 primal cut traits analyzed are summarized in Table 1. Backfat depth exhibited the largest sample size (*n* = 1117), with a mean of 20.4 mm and a standard deviation (SD) of 4.31, indicating moderate variation among individuals. Among the percentage fat traits, butt fat% demonstrated the highest mean value (37.5%) and substantial variability (SD = 4.42), with values ranging from 23.9% to 55.0%. Belly fat% and loin fat% showed comparable mean values (32.6% and 33.6%, respectively) with relatively similar dispersions. Intramuscular fat displayed a mean of 3.87% (SD = 1.20) across a large sample (*n* = 1112), suggesting moderate individual differences. Measurements related to specific anatomical regions, such as ham side fat thickness (mean = 18.0 mm) and shoulder dorsal fat (mean = 21.4 mm), also indicated notable phenotypic variability. In contrast, belly side fat (mean = 2.50 mm, SD = 0.45) exhibited lower mean values and limited dispersion. These descriptive results provide a quantitative overview of phenotypic diversity in economically important fat traits in Canadian commercial pigs, supporting further genetic and genomic analyses.

These traits represent important indicators of carcass composition and meat quality, highlighting their relevance in swine production and breeding programs. Subcutaneous fat, such as backfat and belly fat, significantly contributes to carcass composition and meat quality, while IMF enhances flavor, taste, and palatability of pork [30]. Backfat depth is a key production metric that can be measured in live pigs using ultrasound, serving as a reliable indicator of total carcass fat content and lean meat yield [4]. Modern breeding strategies have prioritized lean carcass traits, leading to improved muscle growth efficiency [6] and reduced fat content, impacting sensory properties, processing potential, and economic value [31]. Fat quality, including firmness, color, shear force, taste, and appearance, plays a vital role in determining meat characteristics and consumer acceptance [32]. Pork belly, a high-value primal cut characterized by subcutaneous and intermuscular fat, remains economically significant, though its fat content has decreased over time due to selection [33]. Other primal cuts, such as shoulder, leg, loin, and belly, also contribute to carcass value, with picnic shoulder, Boston butt, and belly being particularly important for optimizing economic returns [11]. In processing, carcasses are split lengthwise, then cut into primal sections: shoulder, leg, loin, and belly [34]. Subcutaneous fat quality in raw hams, a critical trait for dry-cured ham production, continues to be a focus in certain breeding programs. Traits like fat texture, thickness, and composition influence processing characteristics and consumer appeal, emphasizing the importance of fat quality in modern pig breeding [10].

### 3.2. GWAS and Gene Annotation

**Figure 1:**
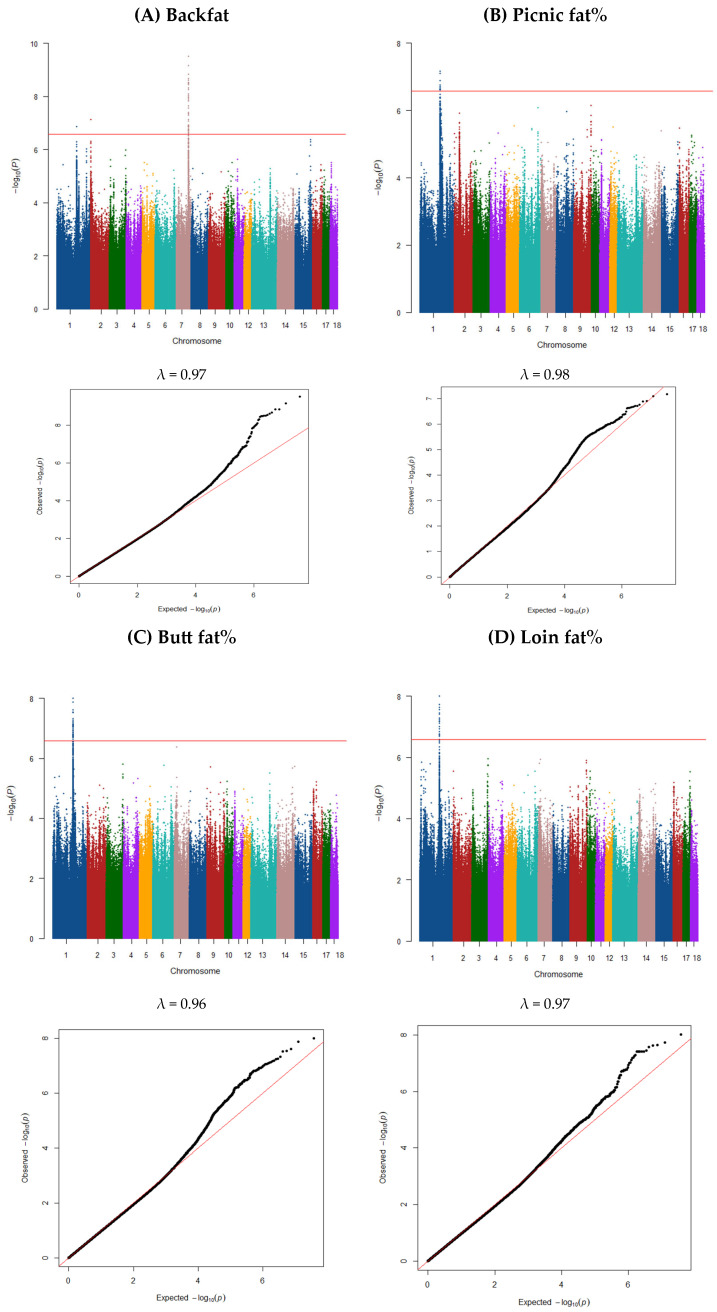

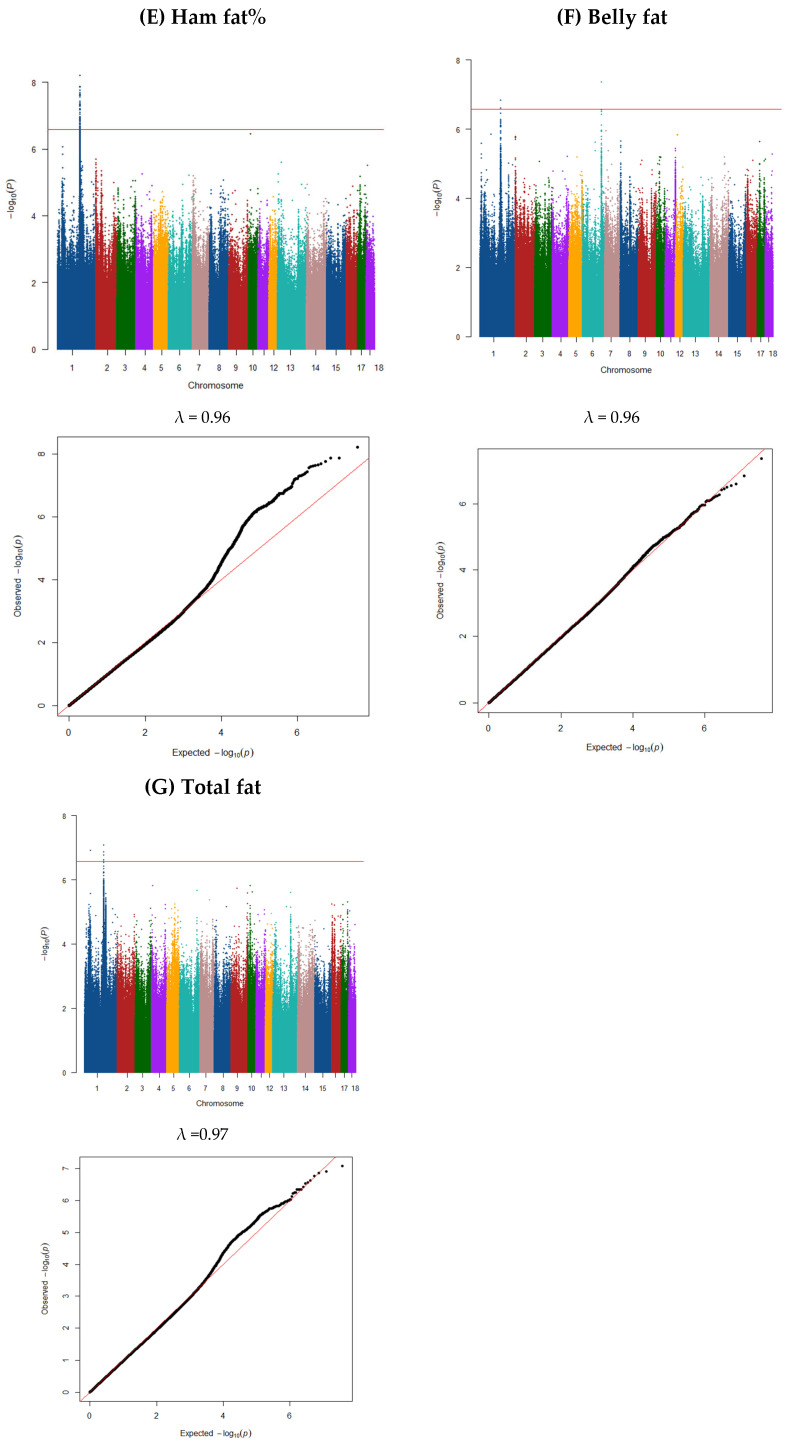
Manhattan and QQ plot of GWAS for individual primal cut traits: (**A**) backfat (λ = 0.97), (**B**) picnic fat% (λ = 0.98), (**C**) butt fat% (λ = 0.96), (**D**) loin fat% (λ = 0.97), (**E**) ham fat% (λ = 0.96), (**F**) belly fat (λ = 0.96), and (**G**) Total fat (λ = 0.97) in commercial pigs. The horizontal red line indicates the genome-wide significance threshold (significant threshold *p* < 2.62 × 10^−7^ correct for multiple testing), we applied corrected for multiple testing using the simple method described by Gao et al. (2008) [24].

**Figure 2:**
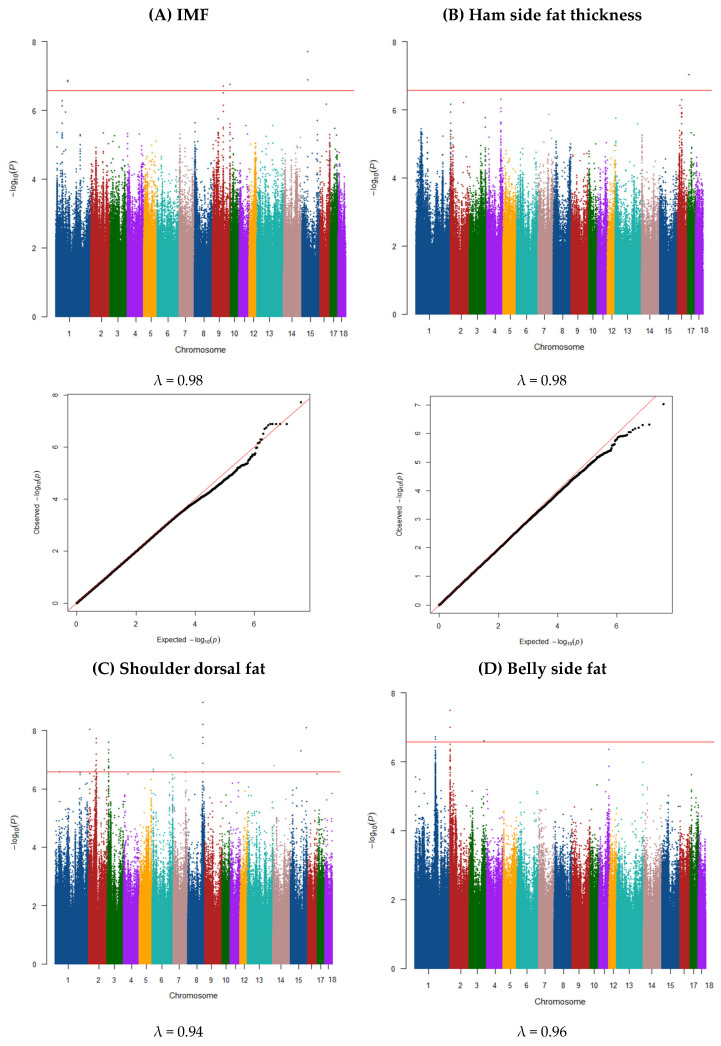

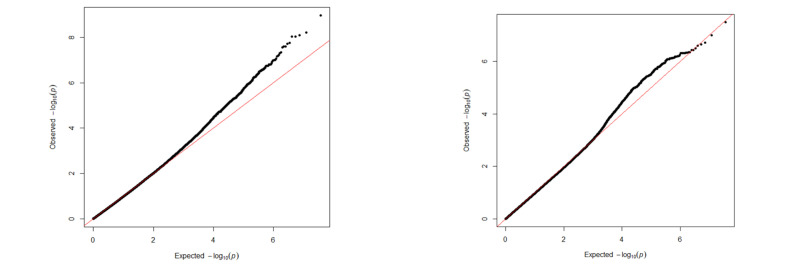
Manhattan and QQ plot of GWAS for individual primal cut traits: (**A**) IMF (λ = 0.98), (**B**) ham side fat thickness (λ = 0.98), (**C**) shoulder dorsal fat (λ = 0.94) and (**D**) belly side fat (λ = 0.96) in commercial pigs. The horizontal red line indicates the genome-wide significance threshold (significant threshold *p* < 2.62 × 10^−7^ correct for multiple testing), we applied corrected for multiple testing using the simple method described by Gao et al. (2008) [24]. Part 2.

Significant SNPs detected based on the GWAS of the 11 traits are summarized in Table 2 and Table 3, with additional details provided in Supplementary Material S1. Candidate genes located near these significant SNPs are also highlighted in these tables and Supplementary Materials. Manhattan and Q–Q plots are shown in Figure 1 and Figure 2. 

### 3.3. Backfat Depth

A total of three QTL were detected for backfat depth (Figure 1A). QLT windows were defined as 0.5 Mb upstream and downstream of groups of significant SNPs in the GWAS that were in <0.5 Mb apart. The most important SNPs in each QTL region are shown in Table 2 and Supplementary Material S1. The window on SSC1 (160.62–161.62 Mb) explained 6% of phenotypic variance for backfat depth and included a single significant SNP. The window located on SSC2 (1.59–2.59 Mb) explained 6% of the phenotypic variance and was also confirmed by one SNP. The third QTL was on SSC7 (96.81–98.12 Mb), which explained the highest proportion of variance for this trait (14%) and contained 58 SNPs.

In total, 111 genes were identified within these windows: 22 on SSC1, 39 on SSC2, and 58 on SSC7 (Supplementary Material S1). Key genes regulating fat deposition, metabolism, and energy balance in these regions include *MC4R*, *IGF2*, *CCBE1*, *PMAIP1*, *CTSD*, and *SHANK2*, among others. Notably, *IGF2* influences muscle growth and fat deposition [35], while *MC4R* plays a role in energy balance and fat mobilization, with polymorphisms linked to backfat thickness [36,37]. *CCBE1* has been linked to backfat thickness [38], and *SHANK2* has been suggested as a strong candidate for regulating backfat [1], and *CTSD* is associated with backfat thickness in pigs [39]. Genes involved in pathways related to lipid metabolism include *SYT8*, which regulates insulin secretion and lipid metabolism [40], and *INS*, which influences glucose and lipid metabolism, affecting fat deposition in pigs [41]. Similarly, *TRPM5* contributes to fatty acid-induced cholecystokinin release [42], and *SLC22A18* impacts fat deposition and lipid metabolism [43]. *CTSD*, *CD81*, and *LPL* play roles in fat metabolism [40]. *ENTPD5* is associated with obesity progression [44], while *ALDH6A1* influences subcutaneous fat deposition and glucose metabolism [45]. Other genes, such as *ABCD4*, contribute to body conformation traits [46], and *PROX2* is linked to backfat thickness in Canadian Duroc pigs [47]. Additionally, *DLST* affects fatty acid accumulation in Laiwu pigs [48].

Genes involved in muscle development and fat-related traits include *TNNI2*, associated with fat percentage and muscle marbling [49], and *LSP1*, which impacts skeletal muscle development [40]. *TNNT3* is linked to muscle fiber development, while *ZNF410* may regulate IMF content [50]. Additional candidates for backfat thickness include *PHLDA2*, which influences glycogen metabolism and fat storage [51], and *OSBPL5*, associated with body composition traits in pigs [52]. Furthermore, *NADSYN1* has been linked to increased backfat thickness [41], while *DHCR7* and *PTGR2* regulate adipocyte differentiation and lipid metabolism [44,53]. Additionally, the microRNA locus ssc-miR-10383 was found to be downregulated in the *longissimus dorsi* muscle of fat-type pigs, influencing IMF deposition and meat quality differences compared to lean-type pigs [54].

The genes and QTLs identified in this study offer further insights into the genetic regulation of backfat traits in pigs. Numerous genes function within overlapping pathways, such as energy balance, lipid metabolism, and muscle development, emphasizing their critical role in fat deposition. For instance, *MC4R* and *IGF2* play central roles in growth and energy balance, making them valuable targets for genetic enhancement efforts. Likewise, genes like *SYT8*, *INS*, and *TRPM5*, which are actively involved in lipid metabolism, provide opportunities for refining fat deposition without negatively affecting lean meat production. The practical implications of these findings for breeding programs are considerable. By utilizing genetic markers associated with backfat thickness and quality, producers can optimize traits like carcass composition, fat quality, and processing efficiency. This not only addresses the demands of the market but also aligns with consumer expectations. Moreover, these genetic insights could lead to improved sensory qualities in pork, such as better marbling and flavor, while minimizing excess fat, resulting in higher product quality and economic benefits.

### 3.4. Picnic Fat%

Genomic regions associated with picnic fat thickness are shown in Figure 1B. There was one significant region at 158.32–160.99 Mb on SSC1 (explained ~9% of the total variance). This region contained 13 SNPs significantly associated with picnic fat (Table 2) and there were 53 genes (Table 2 and Supplementary Material S1) within the window 0.5 Mb upstream and downstream of the significant SNPs. Several of the identified genes are involved in related biological pathways, highlighting their roles in fat deposition and growth traits. For example, *PHLPP1*, which encodes a phosphatase, is a potential candidate gene for average daily weight, backfat thickness, body weight, and carcass weight in pigs [55]. Additionally, *PHLPP1* has been identified as a key contributor to growth and fatness traits in pigs [56]. *CDH20*, involved in the cell adhesion pathway, has been associated with growth and fatness traits in pigs [57]. Studies suggest *KDSR* and related genes could be useful biomarkers for exploring steroid hormone and androstenone biosynthesis in pigs [58]. *Bcl-2* regulates apoptosis of pig adipocytes induced by conjugated linoleic acid (CLA) through the mitochondrial signaling pathway, contributing to reduced back fat deposition in pigs [58].

The identified genes and their associated pathways offer valuable insights into the genetic regulation of picnic fat thickness in pigs. Understanding the roles of genes such as *PHLPP1* and *CDH20* in growth and fatness traits can aid in optimizing carcass composition through targeted breeding strategies. Furthermore, the involvement of *KDSR* and *Bcl-2* in lipid metabolism and adipocyte regulation highlights opportunities to improve fat quality while reducing undesirable fat deposition.

### 3.5. Butt Fat%

Genome-wide association analysis for loin fat identified one QTL (Figure 1C), at 159.13–162.74 Mb on SSC1 (explained ~13% of the total variance), containing 48 significant SNPs associated with the butt fat trait (Table 2). Within 0.5 Mb upstream and downstream of these significant SNPs, 67 genes (Table 2 and Supplementary Material S1) were identified. As these genes (for example: *PHLPP1*, *ZCCHC2*, *TNFRSF11A*, *RELCH*, *PIGN*, *RNF152*, *CDH20*, *MC4R*, *PMAIP1*, *CCBE1*) were common across the traits analyzed in this study, their roles in fat metabolism have been detailed in the earlier sections. Given their recurring roles in fat metabolism, these genes are strong candidates for regulating the butt fat trait in pigs. For instance, *PHLPP1* has been linked to growth and fatness traits, such as backfat thickness, body weight, and carcass weight [55,56]. Óvilo et al. (2006) reported that *MC4R* plays a pivotal role in energy balance and fat accumulation, making it a widely studied gene in breeding programs targeting fat traits [36]. The involvement of these genes in key metabolic pathways highlights their potential for genetic improvement strategies. Hence, the listed genes may qualify as quantitative trait genes candidates for pig butt fat content.

### 3.6. Loin Fat%

GWAS for loin fat identified one QTL (Figure 1D), at 159.67–161.03 Mb on SSC1 that explained ~13% of the total variance, with 31 significant SNPs (Table 2). Within 0.5 Mb upstream and downstream of these significant SNPs, 30 genes (Table 2 and Supplementary Material S1) were identified. Since these genes (*CCBE1*, *CDH20*, *MC4R*, *PIGN*, *PMAIP1*, *RELCH*, *RNF152*, *TNFRSF11A*) were shared among the traits examined in this study, their roles in relation to fat metabolism have been extensively discussed in the previous sections. By incorporating genetic markers linked to the loin fat trait, producers can optimize carcass composition, balancing fat quality and lean meat yield to meet market demands. Furthermore, leveraging insights from previous research, such as the role of *MC4R* and *CCBE1* in fat and growth traits [36,38], can facilitate the development of marker-assisted selection programs.

### 3.7. Ham Fat%

As shown in Figure 1.E, one QTL was identified for ham fat%, at 158.32–161.37 Mb on SSC1 (explained ~14% of the total variance). This QTL contained 73 SNPs significantly associated with ham fat trait (Table 2). There were 59 genes (Table 2 and Supplementary Material S1) within the 0.5 Mb upstream and downstream of the significant SNPs. Key genes such as *PHLPP1*, *ZCCHC2*, *TNFRSF11A*, *RELCH*, *PIGN*, *RNF152*, *CDH20*, *MC4R*, *PMAIP1*, *CCBE1*, and *LMAN1* were previously discussed in this study for their roles in fat metabolism and growth traits. In addition to these recurring genes, other genes identified in this region also contribute to fat metabolism and related traits. For example, SerpinB8, a member of the ovalbumin-like serine protease inhibitor family, plays a role in regulating inflammation in white adipose tissue (WAT) and contributes to the development of obesity [59]. Increased serum levels of *SERPINA12* have been observed in patients with type 2 diabetes [60]. *VPS4B*, involved in endosomal sorting complexes, is essential for transport processes and plays a key role in degrading membrane receptors, regulating both epidermal growth factor and insulin receptors [56]. The *VPS4B*, *PHLPP1*, and *CDH20* genes have been identified as potential candidates for the genetic basis of porcine growth and fatness traits [56].

The identification of this QTL and its associated genes provides valuable insights into the genetic regulation of fat deposition in pigs. Genes such as *PHLPP1*, *CDH20*, and *VPS4B* offer potential for targeted selection to improve fat deposition in ham while maintaining overall carcass quality. Their roles in metabolic pathways, including inflammation regulation and receptor signaling, present opportunities for enhancing both growth efficiency and fat quality through MAS. Moreover, these findings have practical implications for breeding programs aiming to optimize traits like ham fat percentage, which directly affects carcass value and processing quality. For instance, incorporating genetic markers linked to *SerpinB8* and *VPS4B* into breeding strategies could address consumer preferences for balanced fat content while enhancing economic returns for producers.

### 3.8. Belly Fat%

The analysis for belly fat identified two QTL (Figure 1F). The QTL located on SSC1 (159.52–160.73 Mb) explained 7% of the total variance and included one significant SNP. The QTL with the highest percentage of variance was on SSC6 (146.22–147.22 Mb; 14%) and included two significant SNPs (Table 2 and Supplementary Material S1). The regions detected for this trait contained 55 genes (Supplementary Material S1), of which 32 were on SSC1 and 23 were on SSC6.

The analysis for belly fat identified several genes associated with key biological pathways, highlighting their roles in fat deposition and related traits. For example, *TNFRSF11A*, which encodes a protein in the TNF-receptor superfamily [61], activates NFKB during uterine receptivity and early pregnancy and is linked to feed efficiency traits such as daily feed intake [61]. Similarly, *PIGN*, involved in glycosylphosphatidylinositol anchor biosynthesis, facilitates protein attachment to blood cell surfaces and has been associated with feeding behavior and feed efficiency [61,62]. Another notable gene, *CDH20*, a glycoprotein involved in cell–cell adhesion, is associated with variations in growth rates and lean mass percentage in pigs, with SNPs near this gene also linked to obesity [61]. Genes involved in lipid metabolism and fat deposition also play significant roles in regulating belly fat traits. *RELCH* facilitates intracellular cholesterol transport, which is crucial for maintaining cellular lipid homeostasis [63]. *RNF152* regulates mTORC1 signaling, contributing to increased IMF in pigs [64]. These findings are supported by our previous GWAS (Mozduri et al., 2025), which identified overlapping QTLs and candidate genes—such as *CDH20* and *RNF152*—on SSC1 associated with belly fat, firmness, and subcutaneous thickness. This consistency across studies reinforces the biological relevance of these genomic regions in carcass fat regulation [65]. In addition, *LEPR* and *LEPROT* are key genes involved in leptin signaling, regulating feed intake, energy metabolism, and adipocyte lipolysis. Variations in these genes influence fat synthesis, growth, and fat deposition. Furthermore, JAK1 is involved in leptin-induced activation within the adipocytokine signaling pathway, contributing to satiety and energy regulation [66].

Several genes influencing meat quality and fatness traits were also identified. *PDE4B* is associated with both immunity and meat quality traits, particularly fatness traits, and has been shown to interact with *LEPR* [67]. *SGIP1*, linked to energy homeostasis and fat mass in humans, is also associated with backfat thickness in pigs [68]. Similarly, *AK4* plays a critical role in regulating IMF composition, affecting fat metabolism and energy processes in muscle tissue [69]. *DNAJC6* was found to be associated with pH24, a meat quality trait that reflects muscle acidity post-slaughter [67].

Lastly, genes involved in adipose tissue development include *ZCCHC2*, which is downregulated in pig adipose tissue, suggesting its role in regulating fat deposition [70]. These genes, collectively, represent strong candidates for belly fat traits in pigs and provide insights into pathways that can be exploited in breeding programs.

### 3.9. Total Fat%

GWAS for total fat identified two QTLs (Figure 1G), at 51.41–52.41 Mb and 159.57–162.64 Mb on SSC1, explained 6% of the total variance each, and were confirmed by five SNPs in total (Table 2). A total of 82 genes (Supplementary Material S1) were detected, of which 21 and 61 were on SSC1.

Several identified genes play critical roles in lipid metabolism and related traits. For instance, ssc-mir-122, a conserved miRNA, is heavily involved in regulating lipid metabolism [71]. Reduced expression of this miRNA, triggered by a high-cholesterol diet in mini pigs, has been associated with increased body weight and cholesterol levels [72]. Similarly, miR-30a modulates lipid metabolism through its effects on *NEDD4*, influencing macrophage phenotypes and lipid uptake, highlighting its role in adipose tissue development and metabolic regulation [73].

Genes such as *GRP* and *SEC11C* are linked to growth and feeding behavior. *GRP*, a regulatory neuropeptide, influences food intake by stimulating the release of gastrin from gastric G cells. *SEC11C*, essential for protein processing, localization, and secretion, plays a pivotal role in growth and development, with deficiencies leading to severe growth impairments [74]. In addition to metabolic regulators, vascular and physiological processes also play a role in fat deposition. For example, *KCNQ5* is crucial for adipose–vascular coupling in small resistance vessels and contributes to blood pressure regulation, linking cardiovascular and adipose tissue health [75]. Furthermore, *RIMS1* has been directly associated with backfat thickness in Landrace and Yorkshire pigs, reinforcing its potential relevance for total fat traits [76]. The genes identified for total fat traits hold promise for improving economically important traits in pigs through their roles in lipid metabolism, growth regulation, and adipose tissue development. Genes like *ssc-mir-122* and *miR-30a* offer opportunities for optimizing fat deposition via genetic selection, while *GRP* and SEC11C could enhance growth and feeding efficiency.

### 3.10. Intramuscular Fat

Four QTL were detected for IMF (Figure 2A). The window on SSC1 (92.86–93.86 Mb) explained 4% of the total variance and was confirmed by five SNPs, two windows on SSC9 (84.80–85.80 Mb, and 137.966–138.966 Mb) explained 6% and 8% of the total variance, respectively, and were confirmed by one SNP. The other window on SSC15 (44.43–45.43 Mb) explained 5% of the total variance and was confirmed by two SNPs (Table 3 and Supplementary Material S1). A total of 35 genes (Supplementary Material S1) were detected, of which 8 were on SSC1, 11 were on the two regions of SSC9 and 16 genes were on SSC15.

Several of the identified genes are strongly implicated in lipid metabolism and muscle development, making them promising candidates for IMF regulation. For example, *AGMO* plays a critical role in lipid homeostasis and remodeling during adipocyte differentiation, with its expression and activity increasing during this process [77]. Similarly, *ENPP6* regulates lipid and steroid synthesis by controlling the expression of lipid metabolic proteins, highlighting its key role in lipid metabolism [78].

Other genes influence muscle development and fat deposition. *MEOX2*, a transcription factor specific to brown adipose tissue, regulates the differentiation of brown preadipocytes and contributes to muscle-related traits [79]. *CRPPA*, *SOSTDC1*, and *MEOX2* are also associated with variations in muscle development and size, influencing the loin muscle area in Beijing Black pigs [80]. Conversely, FOS negatively regulates IMF formation by suppressing lipid accumulation and downregulating lipogenesis genes such as PPARγ, C/EBPβ, and C/EBPα, as shown in studies on goats [81].

The identified genes offer valuable insights into the genetic regulation of IMF traits, which are essential for meat quality, including marbling and flavor. Genes like *AGMO* and *ENPP6* support lipid metabolism, while *MEOX2* and *SOSTDC1* contribute to muscle development and fat deposition, making them promising targets for genetic selection. 

### 3.11. Ham Side Fat Thickness 

As shown in Figure 2B, there was one signal positioned at 10.43–11.43 Mb on Chr17 (explained ~8% of the total variance). This signal contained one SNP significantly associated with ham side fat thickness (Table 3). There were 18 genes (Table 3 and Supplementary Material S1) within the 0.5 Mb upstream and downstream of the significant SNPs. Several identified genes play crucial roles in lipid metabolism and fat deposition. For instance, *miR-486-5p* regulates fat deposition and lipid differentiation [82], while *ANK1*, part of the ankyrin family, is associated with IMF and meat quality traits such as pork tenderness [82,83]. The *GPAT4* gene plays a role in lipid and glycerophospholipid metabolism, with upregulation in pig livers contributing to increased glycerophospholipid synthesis and lower fatty acid concentrations [84]. Similarly, *NKX6-3* which is linked to higher triglyceride levels, plays a role in lipid metabolism [85]. *SFRP1*, which regulates genes involved in fatty acid synthesis, shows increased expression in obese pigs, further highlighting its role in lipid metabolism [86].

Other genes also contribute to fat regulation and metabolic pathways. For example, IKBKB and NFKBIA are part of the hypothalamic IKKβ/NFκB pathway that influences fat deposition in pigs [87]. *VDAC3* is involved in the ferroptosis pathway, which regulates iron-dependent lipid peroxidation and impacts IMF deposition [88]. The identified genes, such as *miR-486-5p*, *GPAT4*, and *SFRP1*, offer potential for regulating fat deposition and lipid metabolism, optimizing carcass composition and fat quality. Additionally, genes like *ANK1* and *VDAC3* provide opportunities to enhance meat quality traits, including tenderness and marbling.

### 3.12. Shoulder Dorsal Fat

A total of 12 QTLs were identified for this trait (Figure 2C), located at 8.20–9.20 Mb on SSC2 that explained ~11% of the total variance, 53.49–56.06 Mb on SSC2 (~7%), 60.23–64.80 Mb on SSC2 (~5%), 129.12–130.12 on SSC2 (~2%), 10.91–12.14 Mb on SSC3 (~13%), 7.10–8.66 Mb on SSC6 (~2%), 149.42–150.42 Mb on SSC6 (~5%), 167.52–168.52 Mb on SSC6 (~11%), 120.33–121.34 Mb on SSC8 (~6%), 4.12–5.12 Mb on SSC14 (~2%), 83.36–87.36 on SSC15 (~1%), and 127.06–128.06 on SSC15 (~9%). These signals contained 43 SNPs (Table 3) significantly associated with shoulder dorsal fat trait. There were 395 genes (Table 3 and Supplementary Material S1) within the 0.5 Mb upstream and downstream of the significant SNPs. These regions encompassed 395 genes, many of which are implicated in lipid metabolism, fat deposition, and meat quality. For example, *ABHD11* [89] and *ALDH7A1* play roles in lipid absorption and abdominal fat deposition, while *BRD4* supports adipogenesis through *PPARG* expression. *ELOVL1* and related proteins contribute to fatty acid elongation, and *ANGPTL3* regulates lipid metabolism, particularly in controlling fat deposition [89]. Genes like *GTF2IRD1*, *TRIM58*, and *LPL* are linked to IMF content and fat metabolism, which are critical for meat quality [90,91]. *LIMK1* influences meat percentage through its interactions with muscle and meat quality traits [86].

Other genes, including *LGALS12* and *BSCL2*, influence adipogenesis and subcutaneous fat deposition, while *MARK2* and *TMEM38A* affect adipocyte formation and fat accumulation. Additionally, genes such as *DNAJB1*, *DOCK7*, and *NYAP2* are associated with backfat thickness and growth traits [92,93,94]. *ST3GAL3* is involved in lipid metabolism and fatty acid biosynthesis [95]. *SNORDs* influence IMF deposition and adipocyte proliferation [96]. *ADH4* and *ADH7* regulate fatty acid content and IMF in pigs involved in lipid metabolism [97]. These genes, together with *ADH5* and *MTTP*, are key regulators of fatty acid content and composition [97]. Their expression likely influences fat metabolism, particularly IMF content, which affects meat quality in pigs. *MLXIPL*, known for enhancing lipogenesis and fat deposition, is particularly expressed in fatty pig breeds, contributing to their higher fat deposition capacity [98].

### 3.13. Belly Side Fat

A total of three QTLs were identified for this trait (Figure 2D). The window on SSC1 (159.49–160.73 Mb) explained 14% of total variance for this trait and was confirmed by two SNPs. The other windows detected on SSC2 (1.45–2.45 Mb; 14%) and on SSC3 (111.96–112.96 Mb; 8%) were confirmed by two and one SNPs, respectively (Table 3). There were 78 genes (Table 3 and Supplementary Material S1) within the 0.5 Mb upstream and downstream of the significant SNPs. Several identified genes are strongly implicated in lipid metabolism, fat deposition, and meat quality traits. For instance, ssc-miR-10383 is downregulated in the *longissimus dorsi* muscle of fat-type pigs, influencing IMF deposition and meat quality differences compared to lean-type pigs [54]. *PHLDA2* regulates glycogen metabolism and adipose deposition, further emphasizing its importance in fat-related traits [51]. Other genes are involved in cholesterol and fatty acid metabolism. *OSBPL5*, encoding an oxysterol-binding protein, maintains cholesterol balance and influences body composition traits [99], while *NADSYN1* plays a role in glucose regulation and lipid metabolism alongside *INS* and *IGF2*, which are critical for fat deposition and metabolic processes [41,52]. *DHCR7*, located near *IGF2*, has been associated with backfat thickness in various pig populations, further supporting its role in regulating body composition traits [16].

*ABHD1* contributes to lipid breakdown and storage [100], and *EMILIN1* regulates adipogenesis and fat deposition, potentially affecting body size and fat distribution [101]. Several genes are also linked to meat quality. *KCNK3*, which regulates potassium–sodium pumps, is associated with meat tenderness and may influence muscle texture and pork quality [102]. *HADHA* and *HADHB* are involved in fatty acid oxidation, contributing to muscle metabolism and potentially affecting fat deposition and muscle texture [103]. Additionally, *RAB10* influences lipophagy and fat accumulation, highlighting its role in fat metabolism and deposition [104]. The identified genes provide key insights into the genetic regulation of belly side fat traits in pigs. Genes such as *ssc-miR-10383*, *SLC22A18*, and *OSBPL5* can optimize fat deposition and lipid metabolism, while *KCNK3*, *HADHA*, and *HADHB* offer potential to enhance meat quality traits like tenderness and marbling.

### 3.14. SNPs Significantly Associated with Multiple Traits

In this study, we identified several common SNPs associated with multiple traits, suggesting potential pleiotropy. A complete list of key SNPs with pleiotropic effects is provided in Table 4.

**Figure 3:**
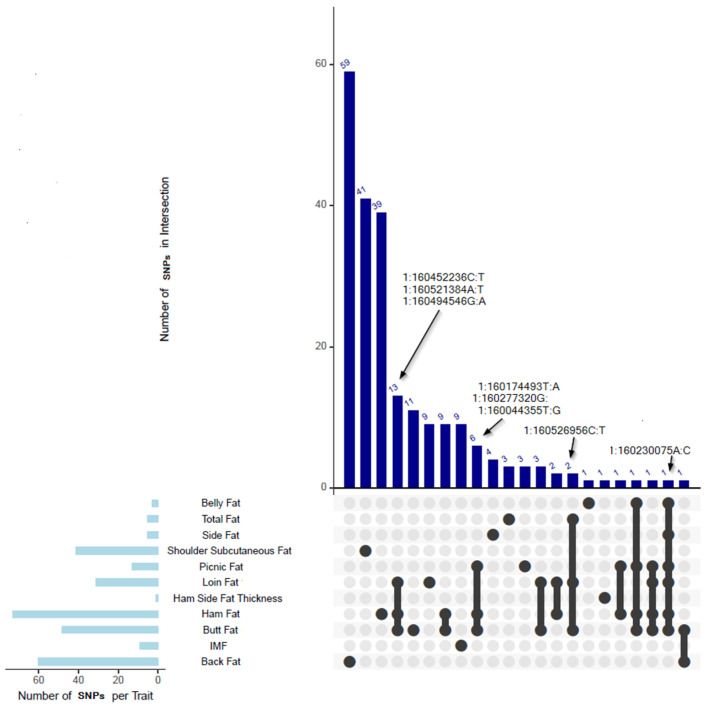
Upset plot showing the number of common SNPs shared among the 11 traits in our GWAS analysis. Notably, SNP *1:160230075A:C* was significantly associated with six fat traits: belly fat, butt fat, ham fat, loin fat, picnic fat, and side fat, and is detailed in Table 4. Two key SNPs were found to be associated with butt fat, loin fat, and total fat; for example, *1:160526956C:T*. In addition, six SNPs—such as *1:160174493T:A*, *1:160277320G:A*, and *1:160044355T:G*—were commonly associated with butt fat, ham fat, and picnic fat. Furthermore, a total of 13 SNPs, including *1:160452236C:T*, *1:160521384A:T*, and *1:160494546G:A* (as examples), were jointly associated with butt fat, ham fat, and loin fat. A complete list of key SNPs with pleiotropic effects is provided in Table 4.

**Figure 4:**
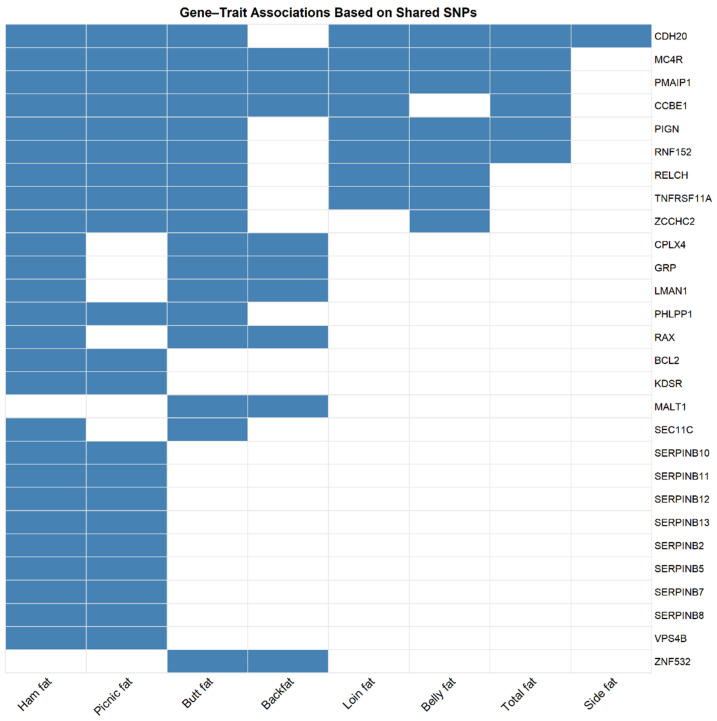
Heatmap illustrating gene–trait associations based on shared SNPs identified in Canadian commercial crossbred pigs. Only genes associated with more than two primal cut traits (i.e., 2, 3, 4, or 6 traits) through shared SNPs are included. Each cell indicates whether a gene (rows) is associated with a given trait (columns), based on overlapping SNPs. Blue cells represent confirmed gene–trait associations, while white cells indicate the absence of association. This visualization highlights pleiotropic genes involved in multiple traits, providing insight into their functional relevance. Gene–trait relationships are derived from the significant SNPs detailed in Table 4, which summarizes mutation types, minor allele frequencies (MAF), genotype distributions, and associated genes for each pleiotropic SNP.

All the SNPs are included in the Supplementary Material S2. Figure 3 shows an upset plot displaying the number of common SNPs shared among the 11 traits in our GWAS analysis. As illustrated in Figure 4, several genes identified through shared SNPs were associated with multiple primal cut traits, reinforcing their pleiotropic roles in adipose tissue deposition. These genes, including *MC4R*, *PIGN*, *CDH20*, and *PMAIP1*, were linked to two or more traits and are summarized in Table 4.

A common SNP located at position 160,230,075 base pairs on the SSC1 region was significantly associated with belly fat, butt fat, ham fat, loin fat, picnic fat, and side fat. The genes associated with this SNP (1:160230075A:C), including *PIGN*, *RELCH*, *RNF152*, *CDH20*, and *MC4R*, were found to be shared among the loin fat and ham fat traits. Additionally, the *CCBE1* gene associated with this SNP (1:160230075A:C) was found to be common to both picnic and butt fat traits. The *CDH20* gene, associated with this SNP, was found to be common to loin fat, picnic fat, butt fat, ham fat, and side fat traits. Moreover, the *PMAIP1* gene linked to this SNP was found to be associated with belly fat, loin fat, and ham fat traits (Table 4).

Another SNP at 160.352 Mbp (1:160352707A:C, SSC1) was significantly associated with butt fat, ham fat, loin fat, and picnic fat. The genes associated with this SNP (1:160352707A:C), including *PIGN*, *RNF152*, *CDH20*, *MC4R*, *PMAIP1,* and *CCBE1*, were found to be shared among the butt fat, ham fat, loin fat, and picnic fat traits. Additionally, an SNP at 160.021 Mbp was significantly associated with belly fat, butt fat, ham fat, and picnic fat. The genes associated with this SNP (1:160021417C:T), such as *CDH20*, *MC4R*, *PIGN*, *RELCH*, *RNF152*, *TNFRSF11A*, and *ZCCHC2* were found to be common to belly fat, ham fat, butt fat, and picnic fat traits. Two SNPs at 160.400–160.526 Mbp (SSC1) were significantly associated with butt fat, loin fat, and total fat (Table 4). A common SNP (1:160526956C:T) located at 160.400–160.526 Mbp (SSC1) was significantly associated with butt fat, loin fat, and total fat. The genes associated with this SNP (1:160526956C:T), included *CCBE1*, *CDH20*, *MC4R*, *PMAIP1*, and *RNF152.* The SNP 1:160400016G:T 1:160400016G (SSC1) at position 160,400,016 base pairs was associated with total fat, butt fat, and loin fat traits. The genes associated with this SNP, including *PIGN*, *RNF152*, *CDH20*, *MC4R*, *PMAIP1*, and *CCBE1,* were found to be common to total fat, butt fat, and loin fat traits (Table 4). Notably, 13 common SNPs located in the SSC1 region between 160.277 and 160.539 Mbp were significantly associated with butt fat, ham fat, and Loin fat, indicating shared genetic influences across these traits (Table 4). Of the 13 associated SNPs, the SNP 1:160277388A:C (SSC1) at position 160,277,388 bp was associated with both loin fat and ham fat traits. The genes associated with these SNPs (1:160277388A:C), including *RNF152*, *RELCH*, *PMAIP1*, *PIGN*, *MC4R*, and *CDH20,* were found to be common to loin fat, ham fat, and butt fat traits. Similarly, another SNP (1:160413164A:T) at position 160,413,164 bp were associated with both loin fat and ham fat traits. The genes associated with these SNPs, including *CCBE1*, *CDH20*, *MC4R*, *PIGN*, *PMAIP1*, and *RNF152*, were found to be common to loin fat, ham fat, and butt fat traits.

Six common SNPs in the SSC1 region from 160.032 to 160.499 Mbp (Table 4) were significantly associated with butt fat, ham fat, and picnic fat. Of the six associated SNPs, the SNP 1:160031812T:A (SSC1) at position 160,031,812 bp, 1:160171880A:G (160,171,880 bp), SNP 1:160044355T:G (position 160,044,355), and 1:160174493T:A (160,174,493 bp) were associated with picnic, ham fat, and butt fat traits. The genes associated with these SNPs include *CDH20*, *MC4R*, *PIGN*, *RELCH*, *RNF152*, and *TNFRSF11A*. Nine SNPs in the SSC1 region from 159.676 to 160.871 Mbp were significantly associated with both butt fat and ham fat (Table 4).

Further analysis revealed an SNP at 161.123 Mbp in the SSC1 region, which was significantly associated with both butt fat and backfat thickness, while another SNP (1:158826231G:C) at 158.82 Mbp was associated with ham fat and picnic fat. Two additional SNPs located between 160.387 and 160.392 Mbp (located on SSC1) were significantly associated with ham fat and loin fat, and three SNPs between 160.176 and 160.235 Mbp were significantly associated with both butt fat and loin fat (Table 4). These results highlight several regions within the SSC1 chromosome that harbor SNPs with potential pleiotropic effects, providing insights into the shared genetic architecture underlying fat deposition traits.

The significant SNPs identified in this study reveal genetic connections among multiple traits, reflecting pleiotropy. These findings corroborate the correlations described in previous studies. For instance, backfat depth, a key indicator of carcass fatness, exhibited strong genetic correlations with traits such as ham fat (0.58–0.79), belly fat (0.64), and loin fat (0.79) [67]. These results suggest that selecting for reduced backfat depth may inadvertently influence other fat-related traits.

Similarly, IMF displayed moderate to high genetic correlations with belly fat (0.76), butt fat (0.75), and total fat (0.72) in our previous study, indicating shared genetic regulation. Traits like belly side fat were also highly correlated (0.74) with the former, showing strong genetic ties to shoulder and ham fat traits. These connections highlight the genetic overlap among primal fat traits, emphasizing the importance of considering correlated traits when designing selection programs.

Notably, the SNPs associated with multiple fat traits, particularly on SSC1, align with reported high genetic correlations among fat content in primal cuts [9]. For example, the overlap of SNPs influencing belly, ham, and loin fat underscores shared genetic architecture, which is beneficial for multi-trait selection. These results support breeding strategies targeting optimal fat deposition in specific regions without compromising overall carcass quality.

To further investigate the shared genetic basis of fat traits, we conducted a meta-analysis integrating GWAS summary statistics from loin fat and butt fat. This analysis revealed a substantial number of significant associations surpassing the genome-wide significance threshold (*p* < 2.62 × 10⁻^7^). Importantly, 20 of these SNPs were also detected in the single-trait GWAS for both traits, supporting the robustness and biological relevance of our findings. These overlapping variants—1:160230075A:C, 1:160352707A:C, 1:160526956C:T, 1:160400016G:T, 1:160277388A:C, 1:160413164A:T, 1:160452236C:T, 1:160521384A:T, 1:160494546G:A, 1:160443956C:A, and 1:160235329T:C, among others—were located near candidate genes previously associated with fat metabolism, including *MC4R*, *PMAIP1*, and *RELCH*. Full details of these shared SNPs are available in Supplementary Material S3.

To validate the robustness of candidate SNPs associated with multiple primal cut traits, linkage disequilibrium (LD) analysis was performed. The results revealed several SNPs in high LD (r^2^ > 0.8), supporting their potential functional relevance. Notably, SNPs such as 1:160230075A:C, 1:160277388A:C, 1:160352707A:C, 1:160526956C:T, and 1:160400016G:T showed strong LD with neighboring markers and were individually associated with multiple carcass fat traits, including loin fat and butt fat thickness. These findings further corroborate their pleiotropic effects and role in the regulation of fat distribution. A full list of high-LD SNP pairs (LD (r^2^ > 0.8)) is provided in Supplementary Material S4. Common genes in windows were associated with multiple traits.

**Figure 5:**
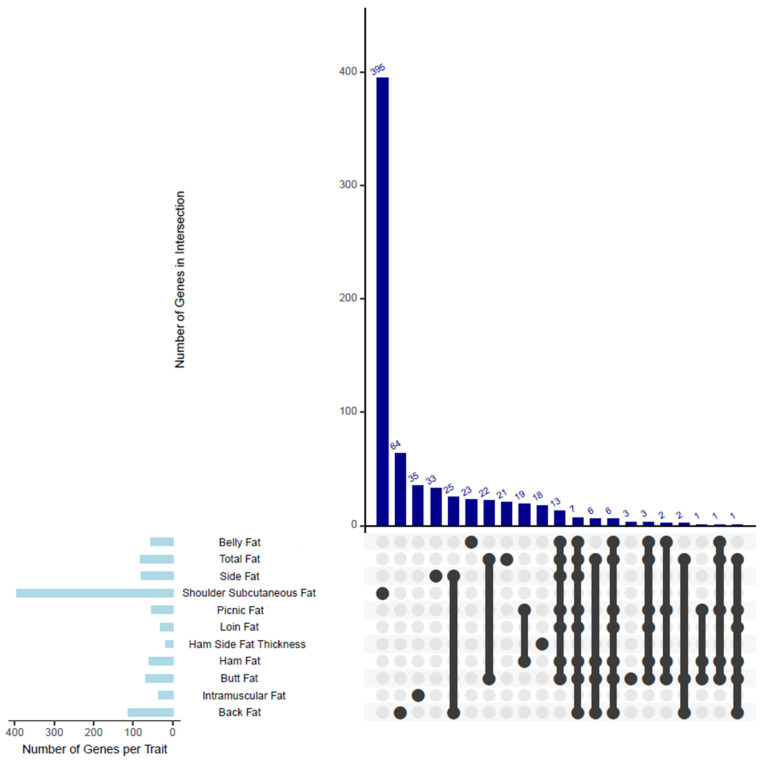
Upset plot displaying the overlapping genes shared among the 11 traits in our GWAS analysis.

The genes that are bolded in the table are common among several traits (Table 2 and Table 3 and Supplementary Material S1). All the genes are included in the Supplementary Material S1. Figure 5 shows an upset plot displaying the overlapping genes shared among the 11 traits in our GWAS analysis. The *MC4R* and *PMAIP1* genes were common for seven traits including back fat, belly fat, picnic fat, butt fat, loin fat, ham fat, and total fat. These two genes were located on SSC1 region 158,32 to 162,74 Mb. The *CCBE1* gene was shared among six traits: back fat, picnic fat, butt fat, loin fat, ham fat, and total fat. It was also located within the SSC1 region, from 158.32 Mb to 162.74 Mb. The genes *LMAN1*, *CPLX4*, *RAX*, *GRP*, *SEC11C*, and *OACYL* were found to be common across four traits: back fat, butt fat, ham fat, and total fat. These genes are located within the SSC1 region, spanning 158.32 Mb to 162.64 Mb. The *ZNF532* and *MALT1* genes were common to three traits: back fat, butt fat, and total fat. These genes were located on the SSC1 region, from 159.13 Mb to 162.74 Mb. The genes *IGF2*, *ASCL2*, *CARS1*, *CD81*, *CDKN1C*, *DHCR7*, *INS*, *KCNQ1*, *NADSYN1*, *NAP1L4*, *OSBPL5*, *PHLDA2*, *SLC22A18*, *Ssc-mir-10383*, *TH*, *TRPM5*, *TSPAN32*, and *TSSC4* were shared between two traits: back fat and belly side fat. These genes were located within the SSC1 region, spanning from 1.452 Mb to 2.59 Mb. The *ZCCHC2* gene was common to four traits: belly fat, picnic fat, butt fat, and ham fat. It is located within the SSC1 region, spanning 158.32 Mb to 162.74 Mb. The genes *TNFRSF11A*, *RELCH*, and *PIGN* were common across six traits: belly fat, picnic fat, butt fat, loin fat, ham fat, and total fat. These genes are located on the SSC1 region, from 158.32 Mb to 162.74 Mb. Finally, the genes *RNF152* and *CDH20* were common to seven traits: belly fat, picnic fat, butt fat, loin fat, ham fat, total fat, and belly side fat. These genes are located on the SSC1 region, spanning 158.32 Mb to 162.74 Mb. These results indicate that primal quality traits are genetically controlled to some extent and can be utilized for selecting specific improvements in the primal and subprimal cuts of the carcass. Genes like *MC4R* and *PMAIP1*, common across seven traits including backfat, belly fat, and ham fat, correspond with high genetic correlations reported between these traits. For example, backfat depth showed strong genetic correlations with loin fat (0.79), ham fat (0.58–0.79), and belly fat (0.64) [67]. These findings suggest that selecting for reduced backfat depth could influence other fat-related traits, a consideration for breeding programs.

Intramuscular fat, which displayed shared SNPs with picnic, butt, and belly fat, also showed moderate to high genetic correlations with these traits (0.61–0.76) in previous studies [62]. Traits like belly side fat, strongly correlated with ham fat area (0.82), align with our findings of shared genes influencing these regions, such as *RNF152* and *CDH20*. Additionally, the genetic correlation between IMF and belly firmness (0.66) highlights the shared influence of genes like *OSBPL5* and *DHCR7*, which regulate lipid metabolism and fat deposition.

These results emphasize the importance of considering correlated traits when implementing selection strategies. Selecting for specific fat traits, such as belly side fat or ham fat, could inadvertently improve overall carcass fat quality due to the high genetic correlations observed across these traits. This shared genetic basis provides opportunities to enhance meat quality traits while maintaining efficiency in breeding program.

### 3.15. Implications for Breeding Programs

This study identifies genes with significant potential for improving primal cuts through precision breeding strategies. Some genes, like *ZNF532*, *MALT1*, *KCNK3*, *HADHA*, and *HADHB*, are trait-specific and can be targeted to enhance individual primal cuts, such as backfat, loin quality, or muscle marbling, without affecting other traits. These genes allow for focused improvements while preserving overall carcass quality. Pleiotropic genes, including *MC4R*, *PMAIP1*, *CCBE1*, *RELCH*, *PIGN,* and *TNFRSF11A* offer opportunities for coordinated manipulation of multiple traits. These genes could reduce fat in undesired regions, such as total and shoulder fat, while maintaining critical quality traits like IMF and belly fat, ensuring balanced improvements across cuts. For breeding programs, strategies include using trait-specific genes for targeted improvements, leveraging pleiotropic genes for multi-trait optimization, and refining selection markers to meet market demands for lean growth, marbling, and firmness. These findings provide a pathway to enhance pork quality and economic value while preserving genetic diversity and efficiency in swine populations.

### 3.16. Functional Enrichment of Candidate Genes

To gain functional insights into the biological relevance of the candidate genes identified through GWAS, Gene Ontology (GO) enrichment analysis was performed across 11 primal cut traits in pigs. The analysis revealed statistically significant enrichment (FDR < 0.1) for four traits—back fat, picnic fat, ham fat, and shoulder dorsal fat—highlighting biologically meaningful processes involved in fat metabolism and regulation. In the back fat trait, candidate genes were significantly enriched in pathways related to small molecule metabolic process (FDR = 0.009), cofactor metabolic process (FDR = 0.009), coenzyme metabolic process (FDR = 0.009), and coenzyme metabolic process. The enrichment of small molecule metabolic process, cofactor and small molecule metabolic processes, and coenzyme metabolic process among candidate genes for backfat thickness is consistent with previous findings in beef cattle. For instance, Martins et al. (2020) identified the same pathway as significantly enriched in Nellore cattle [105], highlighting its relevance to adipose tissue metabolism across livestock species. Genes associated with picnic fat and ham fat were significantly enriched in proteolysis-related processes, such as negative regulation of peptidase activity (GO:0010466; FDR = 6.5 × 10⁻^11^) and cellular protein metabolic process (GO:0044267; FDR = 3.5 × 10⁻^4^). These findings align with previous studies linking protein turnover and protease activity to adipogenesis and fat accumulation in pigs. Notably, candidate genes associated with shoulder dorsal fat thickness were enriched in GO terms related to sensory perception (GO:0007600, FDR = 2.24 × 10⁻^6^), G-protein coupled receptor (GPCR) signaling (GO:0007186, FDR = 2.36 × 10⁻^5^), detection of chemical stimulus involved in sensory perception of smell (GO:0050911, FDR = 1.60 × 10⁻^13^), and detection of chemical stimulus (GO:0009593, FDR = 3.97 × 10⁻^6^).

These results are consistent with previous findings by Blaj et al. (2023), who reported strong enrichment of the same pathways—sensory perception, G-protein coupled receptor signaling, and olfactory signaling—in Piétrain and Large White × Landrace pigs using whole-genome sequence data. In their study, these GO terms were significantly associated with meat-to-fat ratio, a composite trait reflecting carcass composition through the balance between lean muscle and fat content [106]. For the remaining traits—including loin fat, total fat, belly fat, belly side fat, IMF, butt fat, and ham side fat—no GO terms passed the FDR < 0.1 threshold. However, some traits (e.g., belly fat and total fat) showed suggestive enrichment in pathways such as lipid transport, bone remodeling, and ion homeostasis, which may still reflect biologically relevant processes, requiring further validation. Taken together, these enrichment results support the biological plausibility of the candidate genes identified in GWAS and offer new insights into the functional mechanisms underlying regional fat deposition in pigs. The diversity of enriched processes across traits suggests that distinct biological pathways contribute to fat accumulation in different anatomical regions, reinforcing the complexity and trait-specific regulation of adipose tissue development. The full list of enrichment results for all traits is available in Supplementary Material S5.

## 4. Conclusions

This study provides valuable insights into the genetic regulation of carcass and primal fat traits in pigs, identifying significant QTLs and candidate genes through a GWAS with whole-genome sequencing data. By analyzing data from 1118 commercial crossbred pigs, key genes like *MC4R*, *PMAIP1*, *ZNF532*, and *KCNK3* were found to influence fat deposition and quality traits across multiple regions, including backfat, belly fat, loin fat, and ham fat.

Trait-specific genes, such as *ZNF532* and *HADHA*, offer opportunities for targeted improvements in individual cuts, while pleiotropic genes like *MC4R* and *PMAIP1* enable coordinated optimization of multiple traits without compromising IMF or belly quality. These findings provide a framework for designing breeding strategies that balance lean growth with quality traits, aligning with market demands and improving the economic value of pork production. By integrating these genetic markers into genomic selection programs, the swine industry can achieve more precise improvements in carcass composition, enhance meat quality, and develop efficient breeding strategies tailored to consumer preferences.

## Figures and Tables

**Table 1:** Descriptive statistics for individual primal cut traits.

Trait	n	Min	Max	Mean	CV	SD
Backfat depth (mm)	1117	10.3	41.4	20.4	21.16	4.31
Picnic fat%	803	17.4	37.4	26.4	12.61	3.33
Butt fat%	803	23.9	55.0	37.5	11.80	4.42
Loin fat%	802	18.6	51.7	33.6	14.47	4.87
Ham fat%	803	18.2	38.8	27.2	10.91	2.97
Belly fat%	804	18.3	46.3	32.6	12.15	3.96
Total fat%	894	19.4	46.2	31.2	11.90	3.72
IMF (%)	1112	1.49	9.96	3.87	31.00	1.20
Ham side fat thickness	962	5.16	32.2	18.0	20.65	3.71
Shoulder dorsal fat (mm)	964	7.2	58.6	21.4	27.33	5.85
Belly side fat (mm)	1083	0.95	4.00	2.50	17.95	0.45

IMF: intramuscular fat.

**Table 2:** Candidate genes located on significant regions and/or nearby regions for individual primal cut traits in Canadian commercial crossbred pigs. Part 1.

Trait	Chr	Start BP	End BP	SNP (Lowest *p*-Value)	MAF	*p*-Value	Percentage Values	Candidate Genes
Backfat depth	1	160623588	161623588	1:161123588T:C	0.268	1.38 × 10^−7^	0.06	***MC4R***, ***PMAIP1***, ***CCBE1***, ***LMAN1***, ***CPLX4***, ***RAX***, ***GRP***, ***SEC11C***, ***OACYL***, ***ZNF532***, ***MALT1***
Backfat depth	2	1593050	2593050	2:2093050A:G	0.02	7.36 × 10^−8^	0.06	*CTSD*, *SYT8*, *TNNI2*, *LSP1*, *TNNT3*, ***IGF2***, ***ssc-mir-10383***, ***INS***, ***TH***, ***ASCL2***, ***TSPAN32***, ***CD81***, ***TSSC4***, ***TRPM5***, ***KCNQ1***, ***CDKN1C***, ***SLC22A18***, ***PHLDA2***, ***NAP1L4***, ***CARS1***, ***OSBPL5***, ***NADSYN1***, ***DHCR7***, *SHANK2*, *CTTN*
Backfat depth	7	96819123	98121753	7:97619754G:A	0.398	3.11 × 10^−10^	0.14	*ZFYVE1*, *RBM25*, *PSEN1*, *PAPLN*, *NUMB*, *RIOX1*, *ACOT6*, *DNAL1*, *PNMA1*, *MIDEAS*, *PTGR2*, *ZNF410*, *FAM161B*, *COQ6*, *ENTPD5*, *BBOF1*, *ALDH6A1*, *LIN52*, *VSX2*, *ABCD4*, *VRTN*, *SYNDIG1L*, *NPC2*, *ISCA2*, *LTBP2*, *AREL1*, *FCF1*, *YLPM1*, *PROX2*, *DLST*, *RPS6KL1*, *PGF*, *EIF2B2*, *MLH3*, *ZC2HC1C*, *NEK9*, *TMED10*, *FOS*, *JDP2*
Picnic fat	1	158326231	160999409	1:158826231G:C	0.201	6.85 × 10^−8^	0.09	***SERPINB8***, ***SERPINB10***, ***SERPINB2***, ***SERPINB7***, ***SERPINB11***, ***SERPINB13***, ***SERPINB12***, ***SERPINB5***, ***VPS4B***, ***KDSR***, ***BCL2***, ***PHLPP1***, ***ZCCHC2***, ***TNFRSF11A***, ***RELCH***, ***PIGN***, ***RNF152***, ***CDH20***, ***MC4R***, ***PMAIP1***, ***CCBE1***
Butt fat	1	159131461	162741690	1:160021417C:T	0.197	9.99 × 10^−9^	0.13	***PHLPP1***, ***ZCCHC2***, ***TNFRSF11A***, ***RELCH***, ***PIGN***, ***RNF152***, ***CDH20***, ***MC4R***, ***PMAIP1***, ***CCBE1***, ***LMAN1***, ***CPLX4***, ***RAX***, ***GRP***, ***SEC11C***, ***OACYL***, ***ZNF532 MALT1***, ***ALPK2***, ***ssc-mir-122***, ***NEDD4L***, ***ATP8B1***, ***SLC51B***, ***RASL12***, ***KBTBD13***, ***UBAP1L***, *PDCD7*, *CLPX*, *CILP*
Loin fat	1	159676058	161038585	1:160277388A:C	0.129	9.76 × 10^−9^	0.13	***CCBE1***, ***CDH20***, ***MC4R***, ***PIGN***, ***PMAIP1***, ***RELCH***, ***RNF152***, ***TNFRSF11A***
Ham fat	1	158326231	161371456	1:160021417C:T	0.197	6.12 × 10^−9^	0.14	***SERPINB8***, ***SERPINB10***, ***SERPINB2***, ***SERPINB7***, ***SERPINB11***, ***SERPINB13***, ***SERPINB12***, ***SERPINB5***, ***VPS4B***, ***KDSR***, ***BCL2***, ***PHLPP1***, ***ZCCHC2***, ***TNFRSF11A***, ***RELCH***, ***PIGN***, ***RNF152***, ***CDH20***, ***MC4R***, ***PMAIP1***, ***CCBE1***, ***LMAN1***, ***CPLX4***, ***RAX***, ***GRPSEC11C***, ***OACYL***
belly fat	1	159521417	160730075	1:160021417C:T	0.197	1.42 × 10^−7^	0.07	***CDH20***, ***MC4R***, ***PIGN***, ***PMAIP1***, ***RELCH***, ***RNF152***, ***TNFRSF11A***, ***ZCCHC2***
belly fat	6	146226998	147226998	6:146726998T:C	0.05	4.33 × 10^−8^	0.14	*AK4*, *DNAI4*, *DNAJC6*, *DYNLT5*, *JAK1*, *LEPR*, *LEPROT*, *PDE4B*, *RAVER2*, *SGIP1*
Total fat	1	51411474	52411474	1:51911474T:G	0.062	1.22 × 10^−7^	0.06	*B3GAT2*, *KCNQ5*, *OGFRL1*, *RIMS1*, *SMAP1*, *SNORA70*, *ssc-mir-30a*, *ssc-mir-30c-2*
Total fat	1	159576052	162647279	1:160076052G:T	0.064	8.32 × 10^−8^	0.06	***TNFRSF11A***, ***RELCH***, ***PIGN***, ***RNF152***, ***CDH20***, ***MC4R***, ***PMAIP1***, ***CCBE1***, ***LMAN1***, ***CPLX4***, ***RAX***, ***GRP***, ***SEC11C***, ***OACYL***, ***ZNF532***, ***MALT1***, ***ALPK2***, ***ssc-mir-122***, ***NEDD4L***, ***ATP8B1***, ***SLC51B***, ***RASL12***, ***KBTBD13***, ***UBAP1L***

All the genes are included in the Supplementary Material S1. The genes that are bolded in the table are common among several traits. Percentage values: percentage of additive genetic variance explained by each SNP.

**Table 3:** Candidate genes located on significant regions and/or nearby regions for individual primal cut traits in Canadian commercial crossbred pigs. Part 2.

Trait	Chr	Start BP	End BP	SNP (Lowest *p*-Value)	MAF	*p*-Value	Percentage Values	Candidate Genes
IMF	1	92861097	93861130	1:93361097T:C	0.013	1.34E × 10^−7^	0.04	***LOC110256969*** **, *LOC110256971*,**
IMF	9	84800447	85800447	9:85300447T:C	0.032	1.99 × 10^−7^	0.06	*AGMO*, *MEOX2*, *CRPPA*, *SOSTDC1*
IMF	9	137966591	138966591	9:138466591T:C	0.047	1.78E × 10^−7^	0.08	*ENSSSCG00000043470*,
IMF	15	44435007	45435011	15:44935011G:A	0.022	1.93 × 10^−8^	0.05	***DCTD*** **, *WWC2*, *CLDN22*, *CDKN2AIP*, *ING2*, *RWDD4*, *TRAPPC11*, *STOX2*, *ENPP6***
Ham Side fat thickness	17	10434900	11434900	17:10934900G:C	0.022	9.33 × 10^−8^	0.08	*SFRP1*, *GOLGA7*, *GINS4*, *GPAT4*, *NKX6-3*, *ssc-mir-486-2*, *ANK1*, *AP3M2*, *PLAT*, *IKBKB*, *POLB*, *DKK4*, *VDAC3*
Shoulder Dorsal fat	2	8208671	9208678	2:8708671A:G	0.011	8.99 × 10^−9^	0.11	*MARK2*, *SPINDOC*, *PLAAT3*, *LGALS12*, *PLAAT5*, *SLC22A8*, *SLC22A6*, *SLC3A2*, *SNORD26*, *SNORD27*, *SNORD28*, *SNORD22*, *SNORD29*, *SNORD30*, *SNORD31*, *SNORD22*, *U2*, *WDR74*, *TEX54*, *STX5*, *NXF1*, *TMEM223*, *TMEM179B*, *TAF6L*, *POLR2G*, *TTC9C*, *HNRNPUL2*, *BSCL2*, *UBXN1*, *UQCC3*, *CSKMT*, *SNORA57*, *C11orf98*, *INTS5*, *GANAB*, *B3GAT3*, *ROM1*, *EML3*, *MTA2*, *TUT1*, *EEF1G*
Shoulder Dorsal fat	2	53493480	56065282	2:55565282T:C	0.017	1.56 × 10^−7^	0.07	*OR2W3*, *TRIM58*, *OR11L1*,
Shoulder Dorsal fat	2	60239686	64803650	2:62503689T:C	0.017	1.89 × 10^−8^	0.05	*NXNL1*, *TMEM221*, *MVB12A*, *BST2*, *CCDC194*, *PLVAP*, *GTPBP3*, *ANO8*, *DDA1*, *MRPL34*, *ABHD8*, *ANKLE1*, *BABAM1*, *USHBP1*, *NR2F6*, *OCEL1*, *MYO9B*, *HAUS8*, *CPAMD8*, *F2RL3*, *SIN3B*, *NWD1*, *TMEM38A*, *SMIM7*, *MED26*, *SLC35E1*, *CHERP*, *C19orf44*, *CALR3*, *EPS15L1*, *CYP4F55*, *CYP4F22*, *PGLYRP2*, *RASAL3*, *WIZ*, *AKAP8L AKAP8*, *BRD4*, *EPHX3*, *NOTCH3ILVBL*, *SYDE1*, *OR1I1*, *CASP14*, *TEKTL1*, *SLC1A6*, *ADGRE3*, *CLEC17A*, *NDUFB7*, *T ECR*, *DNAJB1*, *GIPC1*, *PTGER1*, *PKN1*
Shoulder Dorsal fat	2	129127496	130127497	2:129627496G:A	0.010	2.23 × 10^−7^	0.02	*GRAMD2B*, *ALDH7A1*, *PHAX*, *SPMIP10*, *LMNB1*, *MARCHF3*
Shoulder Dorsal fat	3	10918189	12142089	3:11441193G:A	0.017	2.51 × 10^−8^	0.13	*MLXIPL*, *VPS37D*, *DNAJC30*, *BUD23*, *ssc-mir-7137*, *STX1A*, *ABHD11*, *CLDN3*, *CLDN4*, *METTL27*, *TMEM270*, *ELN*, *LIMK1*, *EIF4H*, *LAT2*, *RFC2*, *CLIP2*, *GTF2IRD1*, *GTF2I*, *NCF1*, *RCC1L*,
Shoulder Dorsal fat	6	7106710	8667211	6:8167211A:T	0.029	2.06 × 10^−7^	0.02	*PKD1L2*, *GCSH*, *C16orf46*, *ATMIN*, *CENPN*, *CDYL2*, *DYNLRB2*, *MAF*
Shoulder Dorsal fat	6	149424555	150424555	6:149924555A:T	0.010	6.99 × 10^−8^	0.05	*ATG4C*, *DOCK7*, *ANGPTL3*, *USP1*, *KANK4*, *PATJ*
Shoulder Dorsal fat	6	167528502	168528502	6:168028502T:A	0.018	8.59 × 10^−8^	0.11	*ST3GAL3*, *KDM4A*, *PTPRF*, *HYI*, *SZT2*, *MED8*, *ELOVL1*, *CDC20*, *MPL*, *TIE1*, *C1orf210*, *TMEM125*, *CFAP57*, *EBNA1BP2*, *CFAP144*, *OR10AK7H*
Shoulder Dorsal fat	8	120338590	121347226	8:120838590C:G	0.012	1.08 × 10^−9^	0.06	*DDIT4L*, *H2AZ1*, *DNAJB14*, *LAMTOR3*, *DAPP1*, *C4orf54*, *MTTP*, *TRMT10A*, *C4orf17*, *ADH7*, *ADH4*, *ADH5*, *METAP1*
Shoulder Dorsal fat	14	4121916	5121916	14:4621916T:A	0.033	1.59 × 10^−7^	0.02	*LPL*, *SLC18A1*, *ATP6V1B2*, *LZTS1*
Shoulder Dorsal fat	15	86368395	87368395	15:86868395G:C	0.010	4.93 × 10^−8^	0.1	*UBE2E3*, *ITGA4*, *CERKL*, *NEUROD1*, *ITPRID2*
Shoulder Dorsal fat	15	127068134	128068134	15:127568134T:A	0.011	7.99 × 10^−9^	0.09	*NYAP2*,
Belly side fat	1	159498384	160730075	1:160230075A:C	0.182	1.87 × 10^−7^	0.14	***RNF152***, ***CDH20***,
Belly side fat	2	1451972	2455805	2:1951972T:C	0.034	3.19 × 10^−8^	0.14	***IGF2***, ***INS***, ***TH***, ***ssc-mir-10383***, ***ASCL2***, ***TSPAN32***, ***CD81***, ***TSSC4***, ***TRPM5***, ***KCNQ1***, ***CDKN1C***, ***SLC22A18***, ***PHLDA2***, ***NAP1L4***, ***CARS1***, ***OSBPL5***, ***NADSYN1***, ***DHCR7***
Belly side fat	3	111960719	112960719	3:112460719T:C	0.019	2.45 × 10^−7^	0.08	*TCF23*, *PREB*, *ABHD1*, *KHK*, *EMILIN1*, *OST4*, *AGBL5*, *TMEM214*, *MAPRE3*, *DPYSL5*, *CENPA*, *SLC35F6*, *KCNK3*, *CIB4*, *CIMIP2C*, *OTOF*, *DRC1*, *SELENOI*, *ADGRF3*, *HADHB*, *HADHA*, *GAREM2*, *RAB10*,

All the genes are included in the Supplementary Material S1. The genes that are bolded in the table are common among several traits. Percentage value percentage of additive genetic variance explained by each SNP.

**Table 4:** Summary of common SNPs associated with multiple primal cut quality traits in Canadian commercial crossbred pig, including mutation type, minor allele frequency (MAF), genotype frequencies, and associated genes.

SNP ID	Chr	Position (bp)	Associated Traits	Associated Genes and Trait Associations	Mutation Type	MAF	Genotype Frequencies
1:160230075A:C	SSC1	160230075	Belly fat, butt fat, ham fat, loin fat, picnic fat, and side fat	*PIGN*, *RELCH*, *RNF152*, *CDH20*, *MC4R* (Ham fat, Loin fat); *PMAIP1* (Ham fat, Loin fat, belly fat); *MC4R*, *PMAIP1*, *RELCH*, *PIGN*, *RNF152* (Picnic, Butt fat); *CDH20* (Picnic, Butt fat, side fat)	C > A	0.147	24:281:813 (C/C:C/A:A/A)
1:160352707A:C	SSC1	160352707	Butt fat, ham fat, loin fat, and picnic fat	*PIGN*, *RNF152*, *CDH20*, *MC4R*, *PMAIP1*, *CCBE1* (shared across butt fat, ham fat, loin fat, picnic fat)	C > A	0.180	36:330:752 (C/C:C/A:A/A)
1:160021417C:T	SSC1	160021417	Belly fat, butt fat, ham fat, and picnic fat	*CDH20*, *MC4R*, *PIGN*, *RELCH*, *RNF152*, *TNFRSF11A*, *ZCCHC2* (shared across belly fat, butt fat, ham fat, and picnic fat)	T > C	0.1973	43:354:721 (T/T:T/C:C/C)
1:160526956C:T	SSC1	160526956	Butt fat, loin fat, and total fat	*CCBE1*, *CDH20*, *MC4R*, *PMAIP1*, *RNF152* (shared across butt fat, loin fat, and total fat)	T > C	0.1333	20:259:839 (T/T:T/C:C/C)
1:160400016G:T	SSC1	160400016	Total fat, butt fat, and loin fat	*CCBE1*, *CDH20*, *MC4R*, *PIGN*, *PMAIP1*, *RNF152* (shared across Total fat, butt fat and loin fat)	T > G	0.1326	20:257:841 (T/T:T/G:G/G)
1:160277388A:C	SSC1	160277388	Loin fat, ham fat, and butt fat	*CDH20*, *MC4R*, *PIGN*, *PMAIP1*, *RELCH*, *RNF152* (shared across loin fat, butt and ham fat)	C > A	0.1294	19:252:847 (C/C:C/A:A/A)
1:160413164A:T	SSC1	160413164	Loin fat, ham fat, and butt fat	*CCBE1*, *CDH20*, *MC4R*, *PIGN*, *PMAIP1*, *RNF152* (shared across loin fat, butt and ham fat)	T > A	0.1333	20:259:839 (T/T:T/A:A/A)
1:160452236C:T	SSC1	160452236	Loin fat, ham fat, and butt fat	*CCBE1*, *CDH20*, *MC4R*, *PMAIP1*, *RNF152* (shared across loin fat, butt and ham fat)	T > C	0.13026	19:253:846 (T/T:T/C:C/C )
1:160521384A:T	SSC1	160521384	Loin fat, ham fat, and butt fat	*CCBE1*, *CDH20*, *MC4R*, *PMAIP1*, *RNF152* (shared across loin fat, butt and ham fat)	T > A	0.2067	48:367:704 (T/T:T/A:A/A )
1:160494546G:A	SSC1	160494546	Loin fat, ham fat, and butt fat	*CCBE1*, *CDH20*, *MC4R*, *PMAIP1*, *RNF152* (shared across loin fat, butt and ham fat)	A > G	0.1326	20:257:841 (A/A:A/G:G/G )
1:160443956C:A	SSC1	160443956	Loin fat, ham fat, and butt fat	*CCBE1*, *CDH20*, *MC4R*, *PMAIP1*, *RNF152*	C > A	0.1326	20:257:841 (C/C:C/A:A/A )
1:160448259T:G	SSC1	160448259	Loin fat, ham fat, and butt fat	*CCBE1*, *CDH20*, *MC4R*, *PMAIP1*, *RNF152*	G > T	0.1326	20:257:841 (G/G:G/T:T/T )
1:160493051A:G	SSC1	160493051	Loin fat, ham fat, and butt fat	*CCBE1*, *CDH20*, *MC4R*, *PMAIP1*, *RNF152*	G > A	0.1341	20:260:838 (G/G:G/A:A/A )
1:160457673C:G	SSC1	160457673	Loin fat, ham fat, and butt fat	*CCBE1*, *CDH20*, *MC4R*, *PMAIP1*, *RNF152*	C > G	0.1326	20:257:841 (C/C:C/G:G/G )
1:160447734T:C	SSC1	160447734	Loin fat, ham fat, and butt fat	*CCBE1*, *CDH20*, *MC4R*, *PMAIP1*, *RNF152*	C > T	0.1326	20:257:841 (C/C:C/T:T/T )
1:160426503T:C	SSC1	160426503	Loin fat, ham fat, and butt fat	*CCBE1*, *CDH20*, *MC4R*, *PMAIP1*, *RNF152*	C > T	0.1349	20:261:837 (C/C:C/T:T/T )
1:160457667A:G	SSC1	160457667	Loin fat, ham fat, and butt fat	*CCBE1*, *CDH20*, *MC4R*, *PMAIP1*, *RNF152*	G > A	0.1326	20:257:841 (G/G:G/A:A/A )
1:160538585A:G	SSC1	160538585	Loin fat, ham fat, and butt fat	*CCBE1*, *CDH20*, *MC4R*, *PMAIP1*, *RNF152*	G > A	0.1365	21:264:834 (G/G:G/A:A/A )
1:160031812T:A	SSC1	160031812	Picnic, butt fat, and ham fat	*CDH20*, *MC4R*, *PIGN*, *RELCH*, *RNF152*, *TNFRSF11A* (shared across Picnic, butt fat and ham fat)	A > T	0.1926	41:348:729 (A/A:A/T:T/T)
1:160171880A:G	SSC1	160171880	Picnic, butt fat, and ham fat	*CDH20*, *MC4R*, *PIGN*, *RELCH*, *RNF152*, *TNFRSF11A* (shared across Picnic, butt fat and ham fat)	G > A	0.2043	47:364:707 (G/G:G/A:A/A)
1:160044355T:G	SSC1	160044355	Picnic, butt fat, and ham fat	*CDH20*, *MC4R*, *PIGN*, *RELCH*, *RNF152*, *TNFRSF11A* (shared across Picnic, butt fat and ham fat)	G > T	0.1926	41:348:729 (G/G:G/T:T/T)
1:160174493T:A	SSC1	160174493	Picnic, butt fat, and ham fat	*CDH20*, *MC4R*, *PIGN*, *RELCH*, *RNF152*, *TNFRSF11A* (shared across Picnic, butt fat and ham fat)	A > T	0.2020	46:360:712 (A/A:A/T:T/T)
1:160277320G:A	SSC1	160277320	Picnic, butt fat, and ham fat	*CDH20*, *MC4R*, *PIGN*, *PMAIP1*, *RELCH*, *RNF152* (shared across Picnic, butt fat and ham fat)	A > G	0.2012	45:359:714 (A/A:A/G:G/G)
1:160499409A:C	SSC1	160499409	Picnic, butt fat, and ham fat	*CCBE1*, *CDH20*, *MC4R*, *PMAIP1*, *RNF152* (shared across Picnic, butt fat and ham fat)	C > A	0.2028	46:361:711 (C/C:C/A:A/A)
1:160347188T:C	SSC1	160347188	Butt fat and ham fat	*CCBE1*, *CDH20*, *MC4R*, *PIGN*, *PMAIP1*, *RNF152* (shared across butt fat and ham fat)	C > T	0.1996	45:357:716(C/C:C/T:T/T)
1:159676238T:C	SSC1	159676238	Butt fat and ham fat	*CDH20*, *PHLPP1*, *PIGN*, *RELCH*, *RNF152*, *TNFRSF11A*, *ZCCHC2* (shared across butt fat and ham fat)	C > T	0.1801	36:330:752(C/C:C/T:T/T)
1:160539124C:T	SSC1	160539124	Butt fat and ham fat	*CCBE1*, *CDH20*, *MC4R*, *PMAIP1*, *RNF152* (shared across butt fat and ham fat)	T > C	0.2082	49:369:700 (T/T:T/C:C/C)
1:159997967T:A	SSC1	159997967	Butt fat and ham fat	*CDH20*, *MC4R*, *PIGN*, *RELCH*, *RNF152*, *TNFRSF11A*, *ZCCHC2* (shared across butt fat and ham fat)	A > T	0.1950	42:351:725 (A/A:A/T:T/T)
1:160443684A:C	SSC1	160443684	Butt fat and ham fat	*CCBE1*, *CDH20*, *MC4R*, *PMAIP1*, *RNF152* (shared across butt fat and ham fat)	C > A	0.2028	46:361:711 (C/C:C/A:A/A)
1:160246630T:C	SSC1	160246630	Butt fat and ham fat	*CDH20*, *MC4R*, *PIGN*, *PMAIP1*, *RELCH*, *RNF152* (shared across butt fat and ham fat)	C > T	0.2004	45:358:715 (C/C:C/T:T/T)
1:160871456A:T	SSC1	160871456	Butt fat and ham fat	*CCBE1*, *CDH20*, *CPLX4*, *GRP*, *LMAN1*, *MC4R*, *PMAIP1*, *RAX*, *SEC11C* (shared across butt fat and ham fat)	T > A	0.2184	53:382:683 (T/T:T/A:A/A)
1:159675840C:T	SSC1	159675840	Butt fat and ham fat	*CDH20*, *PHLPP1*, *PIGN*, *RELCH*, *RNF152*, *TNFRSF11A*, *ZCCHC2* (shared across butt fat and ham fat)	T > C	0.1801	36:330:752 (T/T:T/C:C/C)
1:160382931T:C	SSC1	160382931	Butt fat and ham fat	*CCBE1*, *CDH20*, *MC4R*, *PIGN*, *PMAIP1*, *RNF152* (shared across butt fat and ham fat)	C > T	0.2012	45:359:714 (C/C:C/T:T/T)
1:161123588T:C	SSC1	161123588	Butt fat and backfat thickness	*CCBE1*, *CPLX4*, *GRP*, *LMAN1*, *MALT1*, *MC4R*, *PMAIP1*, *RAX*, *SEC11C*, *ZNF532* (shared across butt fat and backfat)	C > T	0.2394	64:407:647 (C/C:C/T:T/T)
1:158826231G:C	SSC1	158826231	Ham fat and picnic fat	*BCL2*, *CDH20*, *KDSR*, *PHLPP1*, *PIGN*, *RELCH*, *RNF152*, *SERPINB10*, *SERPINB11*, *SERPINB12*, *SERPINB13*, *SERPINB2*, *SERPINB5*, *SERPINB7*, *SERPINB8*, *TNFRSF11A*, *VPS4B*, *ZCCHC2* (shared across ham fat and picnic fat)	C > G	0.2012	45:359:714 (C/C:C/G:G/G)
1:160391873T:C	SSC1	160391873	Ham fat and loin fat	*CCBE1*, *CDH20*, *MC4R*, *PIGN*, *PMAIP1*, *RNF152* (shared across ham fat and loin fat)	C > T	0.1349	20:261:837 (C/C:C/T:T/T)
1:160386647T:C	SSC1	160386647	Ham fat and loin fat	*CCBE1*, *CDH20*, *MC4R*, *PIGN*, *PMAIP1*, *RNF152* (shared across ham fat and loin fat)	C > T	0.1333	20:259:839 (C/C:C/T:T/T)
1:160196758C:A	SSC1	160196758	Butt fat and loin fat	*CDH20*, *MC4R*, *PIGN*, *PMAIP1*, *RELCH*, *RNF152* (shared across butt fat and loin fat)	A > C	0.1357	21:262:835 (A/A:A/C:C/C)
1:160176058G:C	SSC1	60176058	Butt fat and loin fat	*CDH20*, *MC4R*, *PIGN*, *RELCH*, *RNF152*, *TNFRSF11A* (shared across butt fat and loin fat)	C > G	0.1528	26:290:802 (C/C:C/G:G/G)
1:160235329T:C	SSC1	160235329	Butt fat and loin fat	*CDH20*, *MC4R*, *PIGN*, *PMAIP1*, *RELCH*, *RNF152* (shared across butt fat and loin fat)	C > T	0.1482	25:282:811 (C/C:C/T:T/T)

## Data Availability

Data supporting this study are available from the authors upon reasonable request and subject to approval by the funding organizations.

## Supplementary Materials

The following supporting information can be downloaded at: https://www.mdpi.com/article/10.3390/ani15121754/s1, Supplementary Material S1. Candidate genes located in significant or nearby regions identified in this study through whole-genome sequencing (WGS) for primal cut traits, including back fat, belly fat, total fat, loin fat, ham fat, picnic fat, butt fat, loin intramuscular fat content, ham side fat, shoulder dorsal fat and belly side fat thicknesses in Canadian commercial crossbred pigs. Supplementary Material S2. List of significant SNPs identified in this study. Supplementary Material S3. List of 20 SNPs that were found to be significant in both single-trait and meta-analysis GWAS, providing cross-validation of their relevance. Supplementary Material S4. Linkage disequilibrium (LD) analysis results for SNPs associated with more than two traits, confirming high LD (r^2^ > 0.8) with nearby markers and supporting their selection as candidate loci. Supplementary Material S5. The full list of enrichment results for all traits.
